# Association for Advancement of Science

**Published:** 1853-11

**Authors:** 


American Association for the Advancement of Science.—The want of room in our last issue prevented a notice of the last meeting of this most useful and flourishing Institution. This Association, it is well known, was formed in 1840, under the title of "Association of American Geologists and Naturalists." In 1847 the title was changed to its present one. Since then they have met in many of the most prominent cities of the Union. The last meeting was at Cleveland, O., on the 28th July. Prof. Benj. Pierce, of Cambridge, was elected President, and Dr. Baird, of the Smithsonian Institute, and Prof. St. John, to the offices of perpetual and annual Secretaries. Most of the many papers read were upon strictly scientific subjects, in the several departments of mathematics, physics, natural history, geology, chemistry, geography and ethnology. Dr. Waldo I. Burnett presented a paper upon blood-corpuscle-holding-cells, and their relation to the spleen; and another upon the renal organs and allantois, in vertebrata. Prof. Horsford, of Cambridge, contributed an essay upon the fatal effects of chloroform, which was perhaps of more direct interest to physicians tban any other which was presented. We regret that our space admits of only an abstract of this essay. Prof. H. undertook a long series of careful and elaborate experiments, for the purpose of ascertaining the cause of the occasionally fatal effects of chloroform: whether it be idiosyncracy of temperament on the part of the patient, or want of judgment or care on the part of the physician, or some impurity arising from Fusel oil, an ingredient in cheap alcohol, or, still further, the result of some spontaneous change in chloroform, rendering it unfit for use. He obtained for the purpose of experiment some samples of bad chloroform, and after many various investigations, which are set forth at some length in the report, he arrives at the following—among other—conclusions:

That good chloroform does not spontaneously change in nine months. The ill effects of chloroform are not due to the presence of chlorine, which is such an irritant that it prevents inhalation altogether.

That the ill effects are not due to any poisonous product arising from the action of bleaching salt upon the small quantity of fusel oil in the alcohol employed in the manufacture of chloroform.

That the ill effects *are* due to the peculiarities of constitution of some patients, and in a few rare cases to want of attention or judgment in the person administering it.

The thanks of the medical profession are due Prof. Horsford for these interesting researches: it is only another proof that the rigid cultivation of science is immediately connected with the progress of Medicine, and bears directly in fact, upon the prolongation of human life.

Prof. Riddell, of New Orleans, exhibited his binocular microscope, so contrived that the object may be viewed with both eyes, instead of one, as is usually the case. Objects thus viewed are seen more clearly, the sensation from both eyes being more intense than from one, and the object is also seen not as a flat shadow, but in relief, showing its depth as well as its breadth.

The next annual meeting will be held at Washington, D. C.

---

A Manual of Obstetrics, by Thos. F. Cock, M. D., Physician to the New York Lying-in Asylum, Physician to Bellevue Hospital, &c., S. S. & Wm. Wood, 261, Pearl street, N. Y., 1853, p. 250. 12 mo.

We have received from the publishers the above concise little work, and find it just what it purports to be, a "hand book for medical students attending lectures, a *skeleton collection of facts* to answer as a syllabus for teachers of medical schools." It is designed only as a compend of medical *facts* and is eminently adapted to the wants, not only of teachers and students, but also of the practitioner in his cases of emergency. The classification is natural and the mechanical execution of the work in the usual good style of the publishing House referred to. F.

